# Nomophobia: An Individual’s Growing Fear of Being without a Smartphone—A Systematic Literature Review

**DOI:** 10.3390/ijerph17020580

**Published:** 2020-01-16

**Authors:** Antonio-Manuel Rodríguez-García, Antonio-José Moreno-Guerrero, Jesús López Belmonte

**Affiliations:** Department of Didactics and School Organization, University of Granada, 18071 Granada, Spain; arodrigu@ugr.es (A.-M.R.-G.); ajmoreno@ugr.es (A.-J.M.-G.)

**Keywords:** nomophobia, smartphones, situational phobia, systematic review

## Abstract

This review examines the current literature focused on nomophobia (objectives, methodological design, main variables, sample details, and measurement methods) in the Scopus and Web of Science databases. To this end, we conducted a systematic literature review in accordance with the Preferred Reporting Items for Systematic Reviews (PRISMA) guidelines. The initial sample consisted of 142 articles, of which 42 met the inclusion criteria and were analyzed in detail. The findings show that the current research is in an exploratory phase, with a greater predominance of descriptive, nonexperimental, and cross-sectional studies that explore the prevalence of nomophobia mainly in adolescents and university students. The most widely used measurement instrument is the Nomophobia Questionnaire (NMP-Q) proposed by Yildrim and Correia. In addition, the research suggests that nomophobia negatively affects personality, self-esteem, anxiety, stress, academic performance, and other physical and mental health problems. We are therefore faced with a health problem, which negatively affects a person, causing psychological problems and physical and behavioral changes.

## 1. Introduction

Today’s smartphones present great opportunities and comforts for people; at the same time, they facilitate the accomplishment of tasks and have achieved generalized popularity in the present society [1] thanks to their communicative power and people’s engagement with them [2]. The users of this technology even state that it has become an extension of their body, determining both their identity and their way of being [3].

It is indisputable that these devices have become an integral part of modern life [4] and have come to produce behavioral modifications in everyday habits and actions [5]. The advance of mobile technology, given its ubiquitous nature, has meant that the smartphone has become an indispensable resource in people’s lives [6]. 

However, in recent years, the number of problems arising from the use of smartphones has increased considerably [7]. As a result, the number of investigations into the state of the matter has increased, characterizing this phenomenon as addictive, antisocial, and dangerous [8]. Smartphone addiction is so prevalent that it is already considered to be like any other addiction to harmful substances. Therefore, it is a public health problem [9], which is why, because of the excessive use of this device [10] and the dependence that this technology generates [11], a new pathology known as nomophobia is emerging [12] and being cataloged as a clinical disorder [13].

Nomophobia is seen as a type of contemporary phobia that emerged in the digital age [14,15,16], which is expanding after the integration of the smartphone into society [17]. This term has its origin in England and is the result of the conjunction “non-mobile” combined with “phobia”, that is, fear, anxiety, and discomfort of not having a mobile device at a certain moment or not having access to a device when required [18,19,20,21]. In other words, nomophobia is the fear of feeling disconnected from the digital world [22].

According to [23] or [24], nomophobia is structured in four main dimensions and/or causes: (1) fear or nervousness for not being able to communicate with other people; (2) fear of not being able to connect; (3) fear of not being able to have immediate access to information; and (4) fear of the renunciation of the comfort provided by mobile devices. 

Nomophobia promotes the development of mental disorders, personality disorders [25], as well as problems in people’s self-esteem, loneliness, and happiness [26], especially in the younger population [27,28]. All of this has a great impact on health, which has negative repercussions on other aspects of life such as study and work [29], by creating a strong dependence on mobile technology [30], affecting professional practice by provoking constant distractions [31]. In addition, it is influencing the relationships and interactions between individuals, producing a distance and isolation from the physical world [21].

This modern disorder increases, in turn, the fear of losing immediate access to any information and communication with others [32], which raises the indicators concerning depression, anxiety, anger, aggressiveness [33], stress, nervousness [34], emotional stability [35], and sleep disorders [36].

Likewise, nomophobia presents a direct and significant link with internet use, social network dependence, and anxiety [37]. Due to these factors, it is considered a digital disease, whose risk factor of suffering is increased in the youth population, between 12 and 18 years old [38], and those subjects whose personality tends to be emotionally dependent [39].

In this technological spectrum, the Internet cannot be forgotten as a technology whose access has been enhanced with the expansion of mobile devices. This has caused addictions to both mobile telephony and Internet access. These new addictions, typical of the digital era, tend to proliferate in economically developed regions, where citizens have the resources and means to have the necessary technology [40].

The characteristics of today’s society have caused adolescence to be the most critical age range for suffering from nomophobia, as well as other symptoms such as Internet and video game addiction and the corresponding psychological and emotional implications [41]. Currently, young people are familiar with developing, communicating, interacting, playing, and having fun with other people through digital media. Some young people state that they prefer digital contact to physical contact [42], causing cognitive, behavioral, and physiological alterations [43]. This continuous and abusive action results in the appearance of problems such as a sedentary lifestyle, eating disorders, sleep problems, depression, irritation, aggressiveness, and low self-esteem, among others [44].

As a novelty in the matter, recent studies have focused on measuring the influence of culture on the prevalence of nomophobia. The results show that culture has a relevant role in human behaviors linked to technology [45,46]. However, little has been studied on how culture influences the appearance of nomophobia in people [47].

This study explores the state of the art regarding a very frequent pathology among the youngest population, which has been derived as a consequence of the technological development experienced in contemporary society. It reveals that people in contemporary society are not only addicted to the Internet, videogames, and technology in general, but are also afraid of not having the means and technological resources to perform the basic functions such as relating, communicating, having fun, and accessing information.

We are faced with a very recent problem that is typical of the digital age and caused by the rise of mobile technology in people’s daily lives. For this reason, this research explores the state of the nomophobia issue in scientific research with the greatest impact.

## 2. Method

This review examines existing literature focused on nomophobia in the Scopus and Web of Science databases. In addition, this work shows the main objectives, methodological design, main variables, sample details, and measurement tools of the included investigations. In order to do this, we carry out a systematic review in accordance with the Preferred Reporting Items for Systematic Reviews (PRISMA) guidelines [48] to test the following questions:

RQ_1_ To what extent is nomophobia a growing public health problem?
RQ_2_ To what extent are adolescents more vulnerable to nomophobia than other populations?
RQ_3_ What are the physical and mental health problems and behavioral changes associated with nomophobia?

### 2.1. Search Strategy

During October 2019 we carried out an inquiry that began with the introduction of the keyword “nomophobia” in all possible search fields (title, abstract, keywords, main text, and so on) in both Scopus and the main collection of the Web of Science (WOS, BCI, BIOSIS, CCC, DIIDW, KJD, MEDLINE, RSCI, SCIELO), and contemplating all possible outcomes in the present day. These databases contain most of the current research references and are, in turn, the most consulted by researchers and experts from different areas of knowledge [49]. No limits were given for geographic area, language, year of publication, or method used. We found 149 results (article, meeting, abstract, others). However, the final sample consisted of 42 references.

### 2.2. Inclusion Criteria

**Figure 1:**
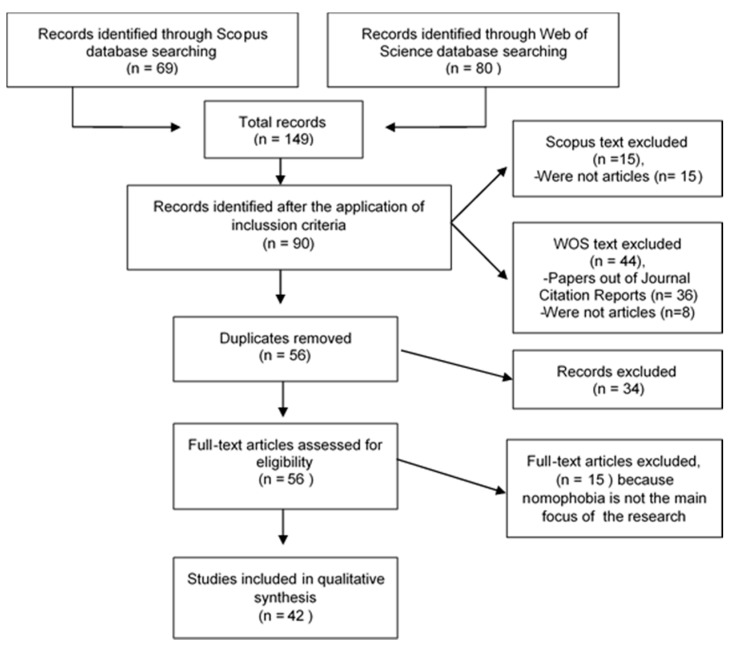
Flow diagram.

The 107 suppressed results were analyzed following a PRISMA protocol for systematic reviews [40]. The main objective was to analyze the articles with the greatest impact that had “nomophobia” as the central focus of their research. In the case of the sample retrieved from the Web of Science (*n* = 80), we deleted references that were not in the Journal Citation Reports indices (*n* = 36) and those that were not articles (*n* = 8) until we reached the final sample. Similarly, we performed this procedure in Scopus, excluding a total of 15 references that did not meet the inclusion criteria with respect to the type of publication. Thus, 91 references were analyzed, 34 of which were deleted as they were articles found in both databases. The 56 remaining records were assessed for eligibility on the basis of the abstract, and in case of doubt, the full text was read. Finally, this figure was reduced to 42 (Figure 1) due to the fact that the main object of study was not nomophobia (*n* = 14) [3,6,8,9,10,21,50,51,52,53,54,55,56,57].

## 3. Results

The articles included in this review were mostly written in English and published between 2010 and 2019. The majority presented results from Turkey (*n* = 8), followed by Spain (*n* = 7) and India (*n* = 6). The most used methods were only quantitative (*n* = 35), nonexperimental (*n* = 26), and cross-sectional studies (*n* = 32). Only *n* = 3 used mixed methods and *n* = 5 were developed with experimental design (Table A1). At the same time, it should be noted that seven of the articles analyzed attempts to create and/or validate a scale to measure nomophobia in different populations (Table A2). Finally, there are three literature review studies (Table A3).

### 3.1. Aims

Most research aims to analyze the prevalence of nomophobia in different groups, whether students, clinic patients, or random people [1,18,19,23,29,30,32,33,36,37]. At the same time, some of them ask if there is any correlation between presenting nomophobia and other alterations of a psychic [5,13,25,26,35,58,59,60] or physical [61] nature, as well as alterations in learning and attention [31,54], in academic performance [2], coping styles [4], or other psychosocial problems arising in the digital and smartphone era [11,15]. Similarly, there are studies that focus on determining risk factors in relation to certain personality traits [16].

### 3.2. Methodological Design

The research analyzed is eminently exploratory, descriptive, nonexperimental, correlational, and cross-sectional, using the questionnaire as a single research collection instrument [1,2,4,13,16,18,19,20,23,25,26,29,30,31,32,33,35,37,39,58,61,62,63,64], presenting a quantitative methodological design. Others, however, extend this information with mixed methodological design, including qualitative techniques, such as interviews or discussion groups [5,20,24,65]. To a lesser extent, some research applies an experimental design, either through the application of cognitive behavioral therapies [5,20] or the use of control and experimental groups [11,13,15,59,62]. Finally, the articles aimed at validating an instrument for measuring nomophobia stand out. They have adapted the tool proposed by Yildrim and Correia [24] to different country contexts, such as Iran [7], Spain [17,27,28], and Israel [14], reaffirming its factors of validity and reliability [12]. Some review articles were also included [17,38,65].

### 3.3. Main Variables

Most research has nomophobia as the main and only study variable [1,4,7,14,17,18,19,22,23,24,27,28,30,31,34,65]. However, other researchers have been interested in examining the relationship between nomophobia and physical factors, such as age [29,33,60], context [29], presence of Carpal tunnel syndrome or median nerve in the wrist [61], and gender [32,33,35,39,58]. Others have studied the relationship between nomophobia and psychic and psychological variables such as anxiety [20,33,37,39], panic disorder [5,20,59], stress [11], depression, avoidance or hostility [12,39], obsessiveness [12,25], FOMO (fear of missing out) [15], personality (extraversion, awareness, emotional stability and regulation, sympathy, and openness to experience) [12,13,16,26,33,35], mindfulness [58], and loneliness and self-happiness [26]. Moreover, others have studied the relationship between nomophobia and sociological, educational, and other factors, including Internet usage and social media [29,37], academic performance [2], learning and attention [13,62], socio-educational variables and collectivism [63], and social threat [11]. 

### 3.4. Sample Details

The samples used in the various studies vary considerably, both in number, where there is a range from one person to 3216, and in the population on which it is focused. First, in relation to the size of the sample, there are studies that have used a sample between one and 150 people [19,21,27,33,59,61], between 151 and 400 people [11,13,18,23,24,25,30,31,34,35,60,62,64], between 401 and 1000 people [1,2,4,12,14,15,16,22,26,29,32,36,37,39,58,63,65], or more than 1000 [7]. Second, with regard to population, studies have focused on volunteers of various classes and types [5,16,20,22,59,60], adolescents or youth [4,7,28,34,35,36], university students where the specialty is not specified [2,12,14,15,23,24,25,26,32,35,39,58,61,62,64,65], university nursing students [18,27,30,31,37], medical students [1,19,29,33], engineering students [29], and professional researchers [11]. In other cases, the population is not indicated, given that the study focuses on a systematic review of papers and journals [17].

### 3.5. Measurement

Most studies have conducted their research exclusively using the Nomophobia Questionnaire (NMP-Q) [1,2,4,7,13,14,18,19,22,24,27,28,29,30,31,32,34,62,64] proposed by [24], which has been adequate and adapted to Persian [7], Indian [2], Spanish [27,28,34], Israeli [14], Italian [12], and Arabic [22] contexts. Other researchers have also added another measuring instrument, such as the Brief Symptom Inventory (BSI) [12,14], Obsessiveness Content Scale (OBS) [25], ultrasonography of the median nerve, Phalen’s test, and reverse Phalen’s test [61], FOMO Scale [15], UCLA Loneliness Scale (ULS-8), Self-Happiness Scale and Rosenberg’ Self-Esteem Scale [26], Stress Scale Social Threat Scale [11], Brief Symptom Inventory [12], Mobile Phone Involvement Questionnaire (MPIQ) [35], Scale of Experiences in Close Relationships (ECR) and Scale of Attention Awareness Mindful-Warning (MAAS) [39], Mindful-Awareness Scale (MAAS) [58], Individualism-Collectivism (INDCOL) [63], Problematic Internet Use Scale (PIUS), Social Appearance Anxiety Scale (SAAS) and Social media use integration scale [37], Emotional Intelligence Questionnaire and Academic iCheating Questionnaire [36], in other scales such as Interviews Scales Inventories Questionnaires [20], Questionnaire to Assess Nomophobia (QANIP), and Temperament and Character Inventory Revised (TCI-R) [16], and in other cases, ad hoc questionnaires of their own elaboration [5,23,33,59,60,65] or ad hoc questionnaires based on NMP-Q [64].

## 4. Discussion

Internet, video games, and now the proliferation of smartphones are causing problems in people’s health [40,41,42,43,44]. The rise of mobile technology as a means of interacting and communicating with people [4,6] has led to the emergence of nomophobia [12], cataloged as a typical pathology of the new millennium [15,16], arising from the constant and abusive use of technology [25], which leads to fear, stress, panic, and anxiety when that technology is not available [11,37,65] for communication or accessing information [32].

The analysis of the 42 articles retrieved from Scopus and Web of Science, which have satisfied the inclusion criteria delimited in the PRISMA review process, leads us to the following inferences. The included literature was mostly recent and in the exploratory phase of research, with a primacy of quantitative and cross-sectional studies with a young population (teenagers and university). The general objective of this current line of research is analyzing the prevalence of nomophobia, as well as the relationship of this disorder with the emergence of problems that affect the psychic, physical, and psychosocial development of the subject, as well as their academic performance.

In relation to the instruments reported from the literature analyzed, it has been ascertained that both the analysis tool initially designed by Yildrim and Correia [24] and its derivatives adapted to other study populations [7,14,22,27,28,34] have proven to be valid and reliable. The initial instrument proposed [24] is therefore consolidated as the most-endorsed tool to analyze nomophobia. 

However, other instruments have also been created based on measurement scales and ad hoc questionnaires that have been used to obtain information on the prevalence of nomophobia and another linked to it, such as those provided by the research of [5,11,12,15,16,23,25,26,33,35,37,39,58,60,64,65], among others.

The results of the analyzed research highlight aspects of gender [26] and young age [33,60] as predictors of nomophobia. As for the students analyzed in the reported studies, especially those in health studies (nursing and medicine) have high levels of nomophobia [1,19,30,31]. In spite of this, engineering students revealed a higher index of this pathology over those of medicine [29]. In the field of education, nomophobia has a negative impact on learning outcomes and academic performance, as has been seen in several studies [2,13,18,62]. This phobia has led to medical and psychosocial disorders such as physical injuries [53] and mental disorders [20,59]. The latter generate a set of fears arising from the non-use of mobile devices [23]. It has also been found that extroverted people and people with deficits in consciousness, attention, emotional stability, and self-esteem are more likely to suffer this pathology [35,39,58].

## 5. Conclusions

Based on the results found in this work, it is concluded that nomophobia is a field of study that is currently in the early stages of research, so that most of the research is in the exploratory phase. Young people exposed to intensive and irrational use of technology are only aware of the advantages it offers and are unaware of the risks they may suffer as a consequence [51]. Therefore, a greater volume of research is needed to explore, investigate, and note which are the most determining variables that influence this contemporary pathology; evidence has shown that nomophobia is closely associated with individual mental health, internet addiction, and behavior modification. In addition, it is necessary to promote efficient and healthy use of mobile technology in learning spaces, in order to avoid the emergence of nomophobia and its consequences.

We, therefore, confirm our three initial hypotheses and can state that nomophobia is a public health problem typical of the digital age and that it is caused by an excessive fear of being without access to a smartphone. The great dependence that the current population has generated towards these devices, due to the different possibilities that they offer, makes them more and more vulnerable, with the adolescent population presenting a greater risk factor. Furthermore, due to the nature of the study and the various scientific contributions published so far, nomophobia is related to the development of personality disorders and mental, physical, educational, and social problems. 

As for the limitations of the present study, there are those of systematic review studies, for which information may have been lost due to not using the correct descriptors or due to the lack of viability of covering all the databases existing at present. However, due to the novelty and specificity of the term, it was decided to introduce only the concept “nomophobia” as a search engine element. As a future line of research, it is proposed to analyze the influence of nomophobia on aspects related to people’s day-to-day life, such as sleeping hours or food.

## Appendix A

**Table A1:** Experimental or nonexperimental studies.

Reference	Country	Aim (s)	Methodology	Sample Details	Main Variables	Measurement	Main Findings
[12]	Italy	The authors hypothesized to find a statistically significant association between nomophobic use of smartphones and psychopathological symptoms as measured by the BSI.	Cross-sectional, descriptive, correlational, and quantitative study with nonexperimental design.	403 volunteers, primarily undergraduate students	Anxiety. Depression. Hostility. Sensitivity. Interpersonal. Obsession-compulsion. Phobic anxiety. Paranoia. Psychosis. Somatization	Nomophobia Questionnaire (NMP-Q). Brief Symptom Inventory (BSI)	The results show that BSI is a reliable and valid instrument with acceptable psychometric properties, and can be administered to nomophobic populations.
[31]	Spain	To analyze the relationship between the level of nomophobia and the distraction associated with smartphone use among nursing students during their clinical practicum.	Cross-sectional, descriptive, correlational, and quantitative study with nonexperimental design	304 nursing students	Nomophobia	Nomophobia Questionnaire (NMP-Q).	Nursing students who show high levels of nomophobia also regularly use their smartphones during their clinical practice, although they also believe it is necessary to implement policies that restrict the use of smartphones while working.
[18]	India	To determine the impact of nomophobia on education among SPPC (students pursuing physiotherapy course).	Cross-sectional, descriptive, correlational, and quantitative study with nonexperimental design	157 nursing students	Nomophobia	Nomophobia Questionnaire (NMP-Q).	Nursing students present nomophobia. There may be a negative impact between nomophobia and academic performance.
[23]	Malaysia	To see the relationship between smartphone usage factors and nomophobia.	Cross-sectional, descriptive, correlational, and quantitative study with nonexperimental design	200 university students	Nomophobia	Ad hoc questionnaire	The fear of the inability to communicate, the fear of loss of connection, the fear of being alone, and the fear of loss of convenience have a significant relationship with nomophobia.
[35]	Spain	To determine the predictive capacity of the criterion variables (extroversion, conscientiousness, emotional stability, agreeableness, openness to experience, self-esteem, age, and gender) in the Spanish version of the MPIQ (Mobile Phone Involvement Questionnaire)	Cross-sectional, descriptive, correlational, and quantitative study with nonexperimental design	242 high school students	Self-esteem. Extraversion. Consciousnes. Emotional stability. Sympathy. Age. Sex Nomophobia	Mobile Phone Involvement Questionnaire (MPIQ).	The results confirm that there is a significant positive predictive relationship between extraversion and nomophobia. A negative predictive relationship was also found between nomophobia and the variable of consciousness. Emotional stability presents a negative predictive correlation. There is a significant negative relationship between self-esteem and nomophobia.
[39]	Turkey	To investigate the mediating effect of mindfulness on the relationship between attachment and nomophobia. In addition, the study also focuses on gender differences in attachment, mindfulness, and nomophobia	Cross-sectional, descriptive, correlational, and quantitative study with nonexperimental design	450 university students	Anxiety. Evasive. Mindfulness. Nomophobia. Gender.	Scale of Experiences in Close Relationships (ECR) Scale of Awareness of Attention Mindfuld-Advertencia (MAAS) Nomophobia Questionnaire (NMP-Q)	In general, people who are emotionally more dependent and crave greater closeness and attention in daily life tend to show higher levels of fear or discomfort when they do not have access to their smartphones. However, gender has a differential impact on the relationship between evasive attachment and nomophobia.
[58]	Turkey	To investigate the impact of individual differences in mindfulness on nomophobia	Cross-sectional, descriptive, correlational, and quantitative study with nonexperimental design	491 university students	Mindfulness. Gender. Nomophobia.	Nomophobia Questionnaire (NMP-Q). Attention Awareness Scale Mindful-Awareness Scale (MAAS)	The results revealed that mindfulness had a significant negative correlation with nomophobia for both men and women. Subjects with lower scores in Attention showed greater anxiety when they were unable to communicate.
[47]	Turkey	This study aims at contributing to literature by investigating the role of espoused culture in influencing nomophobia.	Cross-sectional, descriptive, correlational, and quantitative study with nonexperimental design	490 university students	Nomophobia Individual Collectivism	Nomophobia Questionnaire (NMP-Q) Individualism-Collectivism (INDCOL)	The results suggest that the relationship between vertical collectivism and nomophobia is significant and positive, while the relationship between horizontal collectivism and nomophobia was not significant.
[37]	Turkey	To examine the effect of problematic Internet use, social appearance anxiety, and social media use on nursing students’ nomophobia levels.	Cross-sectional, descriptive, correlational, and quantitative study with nonexperimental design	755 nursing students	Internet usage. Anxiety. Use of social media Nomophobia	Nomophobia Questionnaire (NMP-Q) Problematic Internet Use Scale (PIUS) Social Appearance Anxiety Scale (SAAS) Social media use integration scale	Levels of nomophobia have a strong, positive, and significant relationship with the variables of problematic Internet use, social appearance anxiety, and social media dependence.
[1]	India	To evaluate nomophobia among medical students who are using smartphones.	Cross-sectional, descriptive, correlational, and quantitative study with nonexperimental design	451 medical students	Nomophobia	Nomophobia Questionnaire (NMP-Q)	Medical students suffer from nomophobia, with varying degrees of severity.
[4]	Italy	To explore coping styles implemented in subjects with nomophobia	Cross-sectional, descriptive, correlational, and quantitative study with nonexperimental design	403 jóvenes italianos	Nomophobia	Nomophobia Questionnaire (NMP-Q)	Nomophobic subjects adopt maladaptive coping strategies when faced with stress. Recognition of how nomophobic subjects react provides insight and introduces an approach to preventive and interventional measures in this population.
[33]	Iran	To investigate nomophobia (no mobile phone phobia) among medical students of Islamic Azad University, Tehran Branch	Cross-sectional, descriptive, correlational, and quantitative study with nonexperimental design	100 medical students	Age. Gender. Education. Discomfort. Anxiety. Insecurity.	Ad hoc questionnaire	The study results showed that participants with a lower mean age felt more discomfort, anger, anxiety, and insecurity due to lack of access to smartphones and other related problems compared with other people.
[29]	India	To find out the prevalence of nomophobia among smartphone-using medical and engineering undergraduates of West Bengal and to compare the nomophobic behaviors, predictors, and smartphone usage among them.	Cross-sectional, descriptive, correlational, and quantitative study with nonexperimental design	608 university students (medical 303 and 305 engineering students)	Nomophobia Age. Sex. Residence. Year of study. Socioeconomic variables	Nomophobia Questionnaire (NMP-Q)	Engineering students showed a higher proportion of nomophobes than medical students. A higher proportion of nomophobes between the two groups were women, those with smartphones beyond the age of 2, those with high monthly bills, and those who spend more than 4 hours a day on the smartphone.
[19]	India	To assess the prevalence of nomophobia in medical students.	Cross-sectional, descriptive, correlational, and quantitative study with nonexperimental design	145 medical students	Nomophobia	Nomophobia Questionnaire (NMP-Q)	Nomophobia is prevalent in 1st year medical students.
[36]	France, USA	The author asks two provocative questions: Does Generation Z (Gen Z) adolescents’ emotional intelligence (EI) provoke iCheating? Can emotional intelligence curb nomophobia and thereby mitigate academic iCheating?	Cross-sectional, descriptive, correlational, and quantitative study with nonexperimental design	472 high school students	Nomophobia Emotional Intelligence ICheating	Nomophobia Questionnaire (NMP-Q) Emotional intelligence questionnaire. ICheating academic questionnaire.	Emotional intelligence (EI) directly encourages iCheating but indirectly reduces nomophobia.
[30]	Spain, Portugal	To compare the levels of nomophobia experienced by nursing students at the University of Almeria, Spain and the Polytechnic Institute of Braganza, Portugal.	Cross-sectional, descriptive, correlational, and quantitative study with non-experimental design	258 university students from Spain and Portugal	Nomophobia	Nomophobia Questionnaire (NMP-Q)	The dimensions explored indicate significant levels of nomophobia among both populations of nursing students, with higher levels among the Portuguese population than among the Spanish population.
[63]	Republic of Korea; Hong Kong.	This study explicates nomophobia by developing a research model that identifies several determinants of smartphone separation anxiety and by conducting semantic network analyses on smartphone users’ verbal descriptions of the meaning of their smartphones	Cross-sectional, descriptive, correlational, and quantitative study with nonexperimental design	301 university students.	Nomophobia Anxiety Emotions	Ad hoc questionnaire, based on Yildrim y Correia (2015)	When users perceive smartphones as being extended, they are more likely to connect to devices, which, in turn, leads to nomophobia by increasing the tendency to search for phone proximity. In addition, words related to memory, self, and proximity search are, in fact, more frequent in the high nomophobia group compared with the low nomophobia group.
[60]	Malaysia	Analyze the influence of age on the prevalence of nomophobia	Cross-sectional, descriptive, correlational, and quantitative study with nonexperimental design.	272 people of different ages	Nomophobia Age.	Questionnaire (not specified)	The results determine that young users are more likely to have nomophobia and have a greater risk of it becoming pathological.
[20]	Brazil	To study nomophobia as a manifest behavior that might serve as an indication of a possible anxiety disorder.	Case study. Cognitive behavioral therapy (CBT) with the use of medicines.	Case report (1 person)	Nomophobia Anxiety Panic	Interviews Scales Inventories Questionnaires	Nomophobic behavior produces changes in daily habits and can reveal other aspects to be investigated, such as the presence of comorbid mental disorders.
[59]	Brazil	This study describes the routine use of smartphones and investigates the appearance of possible emotional alterations or symptoms related to their use in patients with panic disorder (PD).	Cross-sectional, quantitative, and descriptive study with experimental design.	50 patients with panic disorders with agoraphobia and 70 controls (volunteers with no psychiatric disorders)	Nomophobia Panic disorder	Ad hoc questionnaire	Both groups exhibited dependence on and were comforted by having a smartphone; however, people with PD and agoraphobia showed significantly more emotional alterations as well as intense physical and psychological symptoms when they were apart from or unable to use a smartphone compared with healthy volunteers.
[5]	Brazil	In this report, the authors present and discuss a hypothesis for the development, in individuals with panic disorder and agoraphobia, of dependence on his or her smartphone.	Case study. Cognitive behavioral therapy (CBT) with the use of medicines.	Case report (1 person)	Nomophobia Panic disorder	Application of evaluation tools (interviews, scales, inventories, and questionnaires).	The patient showed significant medical improvement in his panic disorder and phobias, but there has been no change in his nomophobia.
[25]	USA	To examine the relationship between the Nomophobia Questionnaire (NMP-Q) and the Obsessiveness Content Scale (OBS) of the Minnesota Multiphasic Personality Inventory-2 (the MMPI-2)	Cross-sectional descriptive, correlational, and quantitative study with nonexperimental design.	397 university students	Nomophobia Obsessiveness	Nomophobia Questionnaire (NMP-Q) Obsessiveness Content Scale (OBS)	The findings showed that the OBS latent variable was correlated with all of the four NMP-Q latent variables. Mixed support was found for convergent validity, but high support was found for the divergent validity of the NMP-Q factors. This study contributes to a growing body of literature seeking to better understand the addictive nature of smartphones and takes a new perspective on addiction research and obsessiveness.
[61]	South Korea	To examine the effect of excessive use of smartphones on the carpal tunnel and median nerve in the wrist.	Cross-sectional descriptive, correlational, and quantitative study with nonexperimental design.	125 university students	Nomophobia Carpal tunnel Median nerve in the wrist	Nomophobia Questionnaire (NMP-Q) Ultrasonography of the median nerve, Phalen’s tests, and reverse Phalen’s tests	Excessive use of smartphones may act as a cause to trigger carpal tunnel syndrome due to pressure on the carpal tunnel in the wrist joint; thus, precautions are necessary when using smartphones.
[13]	USA	To examine the impact of different smartphone policies on learning and emotion regulation style	Cross-sectional and quantitative study with experimental design.	160 university students in 4 groups of 40 participants.	Nomophobia Learning Emotion-regulation	Nomophobia Questionnaire (NMP-Q)	Participants who had their smartphone taken away performed best in the test with no other differences. None of the emotional regulation measures moderated the results. These findings provide important insight as to how smartphone policies can optimize learning in the classroom
[62]	USA	To examine how the presence of having a smartphone, the distractibility of text messages, and individual differences in nomophobia might impact learning at different times during a short lecture.	Cross-sectional descriptive, correlational, and quantitative study with experimental design.	160 university students divided into 4 groups of 40 participants.	Nomophobia Learning Attention	Nomophobia Questionnaire (NMP-Q)	Participants who kept their smartphones performed worse on the quiz for material presented in the third quarter of the lecture than those without smartphones. Distracted participants performed worse in the test for the same material than those who were not distracted. Participants higher in nomophobia, especially on subscales having to do with losing connectedness and giving up convenience, performed worse on the quiz for material that occurred in the third quarter of the lecture. Findings indicate that having smartphones in a short lecture has its largest impact on attention and learning 10–15 min into the lecture.
[15]	Turkey	To examine the relationship between nomophobia and FOMO (fear of missing out)	Cross-sectional, descriptive, correlational, and quantitative study with nonexperimental design	538 university students	Nomophobia FOMO	Nomophobia Questionnaire (NMP-Q) FOMO Scale	The results show that a positive moderate level of the relationship was found between nomophobia and FOMO levels
[16]	Spain	The current study set out to establish the relationship between temperament and personality and the development of nomophobia	Cross-sectional, descriptive, correlational, and quantitative study with nonexperimental design	968 participants selected from the Andalusian population.	Nomophobia Temperament Personality	Questionnaire to Assess Nomophobia (QANIP) Temperament and Character Inventory Revised (TCI-R)	The authors found that cooperation is a characteristic that significantly reduces nomophobic levels, particularly for the two factors of smartphone addiction and negative consequences. Furthermore, Reward Dependence appears to be positively related to two of the factors involved in nomophobia, namely smartphone addiction and loss of control, suggesting a relationship between nomophobia and personality.
[26]	Turkey, Pakistan	This study focused on examining the prevalence of nomophobia among university students; and the relationship between nomophobia, self-esteem, loneliness, and self-happiness with respect to gender and year of study of the university students in Pakistan and Turkey.	Cross-sectional, descriptive, correlational, and quantitative study with nonexperimental design	729 university students from Turkey and Pakistan.	Nomophobia Self-esteem Loneliness Self-happiness	Nomophobia Questionnaire (NMP-Q) UCLA Loneliness Scale (ULS-8) Self-Happiness Scale Rosenberg’ Self-Esteem Scale	According to multivariate effects results, the main effect of gender on self-esteem and nomophobia was statistically significant, which indicates that differences between male and female students with respect to self-esteem and nomophobia were significant. The study demonstrated differences between Turkish and Pakistani students’ scores on nomophobia, loneliness, and self-happiness were significant, while differences in self-esteem across countries were not statistically significant.
[2]	India	To assess the pattern of usage of smartphones and its effects on the academic performance of students	Cross-sectional, descriptive, and quantitative study with nonexperimental design	554 university students	Nomophobia Academic performance	Nomophobia Questionnaire (NMP-Q) adaptation	The pattern of usage of smartphones among students showed alarming indication that students have been addicted to a smartphone, which, in turn, affects their academic performance in a negative way
[64]	Peru	To identify symptoms that have not yet been detected by intensive smartphone use	Cross-sectional, descriptive, and mixed methods with nonexperimental design	461 university students	Nomophobia	Focus group Ad hoc questionnaire	Three symptomatic factors of nomophobia were identified: feelings of anxiety, compulsive smartphone use, and feelings of anxiety and panic.
[11]	Canada	This study examined the process by which nomophobia’s effect on stress unfolds	Cross-sectional descriptive, correlational, and quantitative study with experimental design	270 young business professionals divided into four groups.	Nomophobia Stress Social Threat	Nomophobia Questionnaire (NMP-Q) Stress Scale Social Threat Scale	The authors found that nomophobia leads to stress via social threat when uncertainty or lack of control are present. Only under the condition of low uncertainty and high control does nomophobia not lead to stress.
[32]	Turkey	To investigate the prevalence of nomophobia among young adults in Turkey	Cross-sectional descriptive, correlational, and quantitative study with nonexperimental design.	537 university students	Nomophobia Gender	Nomophobia Questionnaire (NMP-Q)	The results revealed 42.6% of young adults had nomophobia, and their greatest fears were related to communication and information access. The study also found that gender and the duration of smartphone ownership had an effect on young adults’ nomophobic behaviors, whereas age and the duration of smartphone ownership had no effect

**Table A2:** Adaptation and validation of questionnaires to measure nomophobia.

Reference	Country	Aim (s)	Methodology	Sample Details	Main Variables	Measurement	Main Findings
[14]	Israel	Translate and validate the Nomophobia Questionnaire (NMP-Q created by Yildrim and Correia (2015).	Quantitative method, of correlational character.	403 volunteers, primarily undergraduate students (no specialty specified)	Nomophobia	Nomophobia Questionnaire (NMP-Q)	The Italian version of NMP-Q has proven to be viable.
[22]	Kuwait	Develop and validate the Arabic version of the NMP-Q questionnaire	Quantitative method.	512 Kuwaiti volunteers with an average age of 20 years	Nomophobia	Nomophobia Questionnaire (NMP-Q)	The Arabic version of NMP-Q has proven to be consistent and reliable.
[34]	Spain	Adapt the NMP-Q questionnaire to Spanish	Quantitative method, of correlational character.	306 students from Navarra, Asturias, and Salamanca	Nomophobia	Nomophobia Questionnaire (NMP-Q)	The Spanish version of the nomophobia questionnaire (NMP-Q) was found to be valid and reliable for assessing nomophobia.
[27]	Spain	Translate the original (written in English) version of the Nomophobia questionnaire, adapting it culturally to the Spanish sociolinguistic context, and analyze the psychometric properties of the Spanish version with a sample of nursing students.	Quantitative method, of correlational character	65 nursing students and 20 subject matter experts	Nomophobia	Nomophobia Questionnaire (NMP-Q)	The results of this study mean that a suitable tool can be applied to nursing professionals with the aim of facilitating the diagnosis of addictive behaviors in relation to the mobility of telephone use.
[7]	Iran	This study aimed to confirm the construct validity of the Persian NMP-Q using Rasch and confirmatory factor analysis (CFA) models.	Cross-sectional and quantitative study.	3216 Iranian adolescents	Nomophobia	Nomophobia Questionnaire (NMP-Q)	The authors concluded that the Persian NMP-Q can be used to assess nomophobia among adolescents. Moreover, NMP-Q users may compare their scores between genders in the knowledge that there are no score differences contributed by different understandings of NMP-Q items.
[28]	Spain	Adapt and validate the scale to measure nomophobia (NMP-Q) to the Spanish context.	Cross-sectional and quantitative study.	372 estudiantes de ESO.	Nomophobia	Nomophobia Questionnaire (NMP-Q)	The results allowed the validation of the Yildirim and Correia scale.
[24]	Turkey	This study sought to contribute to the nomophobia research literature by identifying and describing the dimensions of nomophobia and developing a questionnaire to measure nomophobia.	Mixed methods with exploratory design.	301 university students	Nomophobia	Interview Nomophobia Questionnaire (NMP-Q)	Four dimensions of nomophobia were identified: not being able to communicate, losing connectedness, not being able to access information and giving up convenience. The NMP-Q was shown to produce valid and reliable scores; and thus, can be used to assess the severity of nomophobia.

**Table A3:** Literature review.

Reference	Country	Aim(s)	Methodology	Main Findings
[38]	Cyprus	To examine the types of digital diseases arising from the use of social media and problematic use as a result of digitization, including nomophobia, cyberchondria, and the fear of getting lost. In English they are nomophobia; cyberchondria; FOMO (fear of missing out).	Literature review	The addiction takes them to a dead end, causing the appearance of digital diseases that lead to various psychological disorders in individuals. Additional studies on digital diseases would provide essential data on their symptoms in individuals. An overview of the literature shows that most studies examine nomophobia, FOMO, and cyberchondria as new diseases.
[65]	Italy	To have an overview of the existing literature, discussing the clinical relevance of this pathology, its epidemiological characteristics, the available psychometric scales, and the proposed treatment.	Literature review	The link between the new technologies and their psychopathological impact is not yet clear, and more research is needed in this field.
[17]	India	To provide clarity on the social cognitive effects of screen addiction, which leads to nomophobia among teenagers, and to become better informed as a researcher in order to inform others of best practices and solutions with regard to new media technology consumption	Literature review	The level of addiction has an influence on the environment with which the youth have connected. The level of addiction stands high among hostel students in many cases. Male and female respondents stand almost equal in many research articles. The present study finds out whether the teens are addicted to screen and smartphones or not and the aspects which made them use the same platform.
